# Efficacy of botulinum toxin and surgery in managing acute acquired comitant esotropia

**DOI:** 10.1186/s40662-025-00431-7

**Published:** 2025-04-01

**Authors:** Xiangxiang Liu, Jiayu Chen, Jie Hao, Zhaojun Meng, Weibin Chen, Huijian Li, Jing Fu

**Affiliations:** Beijing Tongren Eye Center, Beijing Tongren Hospital, Beijing Key Laboratory of Ophthalmology and Visual Sciences, Capital Medical University, Beijing, 100730 China

**Keywords:** Acute acquired comitant esotropia, Botulinum toxin, Strabismus surgery, Anisometropia

## Abstract

**Background:**

Acute acquired comitant esotropia (AACE) can significantly impair binocular vision, and its prevalence is increasing. This study aims to compare the effectiveness of botulinum toxin (BTX) injections with strabismus surgery in patients diagnosed with AACE and to investigate the factors predicting success.

**Methods:**

Sixty AACE patients were included in this prospective comparative clinical study. Twenty-seven patients underwent incisional strabismus surgery (surgery group) and 33 patients received BTX injection (chemodenervation group). Patients were followed up visit at 1, 3, and 6 months post-treatment. The primary outcome was the success rate at 6 months post-treatment, defined as a horizontal deviation of 10 prism diopters (PD) or less with confirmed binocular single vision. Secondary outcomes included risk factors for the recurrence of AACE.

**Results:**

The present study included 27 patients in the surgery group and 33 in the chemodenervation group. No significant differences were observed in the motor success rate at 1 and 3 months post-treatment between the two groups. However, the surgery group had a significantly higher motor success rate compared to the chemodenervation group at 6 months post-treatment (100% vs. 69.7%, *P* < 0.001). The success rate of achieving stereopsis at near ≤ 100 arcsec in the chemodenervation group was significantly higher than in the surgery group at 1 month post-treatment (51.5% vs. 14.8%, *P* < 0.001). By the 6 months post-treatment, no significant differences were observed in sensory outcomes between the chemodenervation and surgery groups (*P* > 0.05 for all). In the chemodenervation group, patients with an anisometropia less than 1 dioptor (D)demonstrate significantly higher motor success rate, and better sensory outcomes, including stereopsis at near (84%, 21/25) and stereopsis at near ≤ 100 arcsec (56%, 14/25), compared to those with anisometropia of 1 D or more (*P* = 0.044).

**Conclusion:**

The success rate after BTX injection was similar to that of surgery for 3 months but lower at 6 months post-treatment. Patients who received BTX showed restoration of stereopsis within the initial first postoperative month, with sustained preservation of this function across 6 months post-treatment. Anisometropia of 1 D may indicate suitability for BTX injection as a preferred treatment option for AACE.

*Trial registration* This study was registered on the Chinese Clinical Trial Registry (ChiCTR2100053717. Registered 28 November 2021. https://www.chictr.org.cn/showprojEN.html?proj=140975).

## Background

Acute acquired comitant esotropia (AACE) is a rare but increasingly recognized type of strabismus characterized by a sudden onset of a comitant esodeviation, typically occurring without neurological or systemic abnormalities [1]. The prevalence of AACE, traditionally historically estimated at around 0.3%, has been reportedly increased to 2.4% in younger populations, probably due to excessive smartphone use and prolonged near work [2, 3]. This increasing incidence in cases underscores the importance of awareness, as AACE can significantly impair binocular vision and depth perception, essential for daily activities such as reading and driving [4]. Without timely intervention, the condition may lead to chronic visual disturbances and diminished quality of life, highlighting the critical need and demand for early diagnosis and treatment [5].

Incisional strabismus surgery is commonly regarded as the standard treatment for AACE, providing stable ocular alignment [6]. In addition, botulinum toxin (BTX) has emerged as a promising alternative for managing strabismus [7]. Recent studies have explored both surgical interventions and BTX injections in the management of AACE, with surgery being considered the standard treatment due to its consistent and stable alignment outcomes. However, BTX has gained attention as a minimally invasive alternative, offering quicker recovery and reduced risks of tissue damage. While these treatments have been compared in terms of overall success rates and motor outcomes, gaps remain in understanding key factors influencing long-term efficacy and sensory outcomes.

This study provides additional insights into the effectiveness of BTX in managing AACE, aiming to evaluate its outcomes in comparison to surgery and to identify predictors of optimal treatment outcomes. This comparative study is crucial for informing clinical practice and enhancing personalized care for patients, especially in patients with anisometropia. By emphasizing this aspect, we hope to clearly differentiate our study's objectives from existing research and contribute to evidence-based decision-making and optimize treatment strategies for AACE.

## Method

This prospective, comparative clinical study was registered on the Chinese Clinical Trial Registry (ChiCTR2100053717) and approved by the medical ethics committee of Beijing Tongren Hospital (No. TREC2022-KY020). The study adheres to the tenets of the Declaration of Helsinki. Written informed consent was obtained from all participants or their parents for those under the age of 18 years. We included patients diagnosed with AACE who were treated at the Department of Strabismus and Pediatric Ophthalmology, Beijing Tongren Hospital, from January 2022 to January 2024.

AACE was defined by a sudden inward deviation of the eye and/or the onset of diplopia, either observed by the patient or caregiver due to a noticeable change in eye alignment. The inclusion criteria included a confirmed date of deviation onset, evaluation within 6 months of its onset, and a minimum follow-up period of 6 months post-treatment. In addition, participants should also meet the following criteria: (1) esotropia distance deviation ≥ 20 prism diopters (PD); (2) monocular best-corrected visual acuity (BCVA) of 1.0 or higher; (3) normal eye movement in each direction; (4) horizontal diplopia with an equal separation of images in all directions; and (5) a certain binocular visual function. Patients were excluded if they had a prior history of strabismus, intraocular surgery or trauma, relevant pathological findings on neuroimaging, neurological or developmental disorders, an incomitant deviation, or abnormal eye movements.

Participants were divided into two groups: the surgery group, which received traditional incisional strabismus surgery, and the chemodenervation group, which received BTX injection. Treatment decisions were made based on patients’ preferences after discussing the advantages and disadvantages of each option. In the surgery group, bilateral medial rectus muscle recession was performed following standardized protocols [8]. For deviations ≤ 25 PD, a recession of 4.0 mm was performed. For each subsequent increase of 5 PD, the recession was increased by 0.5 mm, up to a maximum of 5.5 mm for deviations between 35 and 40 PD. In the chemodenervation group, botulinum toxin-A (BTX-A, BOTOX^®^, Allergan, Inc., Irvine, California, US) was administered to the bilateral medial rectus muscles without conjunctival incision. The procedure involved abducting the eye, positioning the needle tip at the plica semilunaris, penetrating the conjunctiva, advancing the needle approximately 1.5 cm along the muscle path, and injecting BTX-A. The injections were performed under a microscope to ensure precise needle placement. For younger children or patients unable to cooperate, general anesthesia was used prior to the procedure. Although standardized tables correlating BTX dosage with specific angles of deviation were not available, patients with a deviation of less than 30 PD received 2.5 IU per 0.1 mL of BTX-A, while those with a deviation of equal to or more than 30 PD were administered 5 IU per 0.1 mL of BTX-A. One experienced surgeon (J.F.) performed all procedures.

All participants underwent comprehensive ophthalmic examinations including BCVA, refractive status, accommodative and convergence test, and assessment of anterior and posterior segment. Cycloplegic refraction was conducted using a standardized protocol for all patients [9]. After 30 min, if the pupil showed dilated and no reaction to light, the refractive errors were measured. Spherical equivalent (SE) was defined as the sum of the spherical power and half the cylindrical power. The stimulus  accommodative convergence/accommodation (AC/A) ratio was assessed using the synoptophore method following prism and alternate cover testing. Deviation was first measured with fully corrected spectacles, then re-measured with − 3.0 D lenses added. The AC/A ratio was calculated by dividing the difference in deviation between the two conditions by − 3.0 D [10].

Motility examinations assessed ductions, versions, and prism cover tests at 6 m and 33 cm. Fusion and stereoacuity at distance fixation were measured using synoptophore testing, while stereoacuity at near fixation was evaluated with the random dot stereograms test. Additional data collected during the initial visit included the duration of esotropia, and any systemic comorbidities. All patients underwent head magnetic resonance imaging or computed tomography scans to exclude nervous system disorders, with the imaging showing a normal course of the extraocular muscles.

The angle of deviation, fusion, and stereopsis data were documented pre-treatment and at 1, 3, and 6 months post-treatment, respectively. To ensure that the effects of the BTX had fully dissipated, a follow-up period of 6 months was selected. The primary outcome measure was the difference in success rates at 6 months post-treatment. Motor success was defined as a horizontal deviation of ≤ 10 PD, measured using the prism and alternate cover test at distance or near. Sensory success was defined as the presence of any evidence of sensory fusion or stereopsis at follow-up visits.

Secondary outcomes included the angle of deviation, stereoacuity, and factors predicting success rate at 6 months post-treatment. The effective rate and the proportion of patients achieving fusion and stereopsis were compared between the two groups. Clinical factors influencing motor success were analyzed, including age, SE, anisometropia as well as pre-treatment near and distance deviation angles. Deviation angles in patients with anisometropia were recorded separately at near and distance fixation to assess the potential impact of refractive asymmetry on motor success. Subgroup analysis was performed based on anisometropia levels (< 1 D or ≥ 1 D) and deviation angles. Complications including ptosis, vertical strabismus, muscle slippage, and conjunctival cyst formation were monitored during follow-ups.

Statistical analysis was performed using SPSS version 23.0 for Mac (IBM). Normality was determined by the Shapiro–Wilk test, and data following a normal distribution are presented as mean ± standard deviation; data that do not follow a normal distribution are presented as median. The independent samples *t*-test was applied to compare continuous variables between two groups. Repeated measures ANOVA was used to test the variance of pre- and post-treatment near and distant deviation angles through 6 months. Categorical variables were presented as frequencies and percentages, with the Fisher’s exact test used to assess differences in sex ratio and success rate between the surgery and chemodenervation groups. For each analysis, a two-tailed model was utilized, and *P* < 0.05 indicated that the difference was considered statistically significant.

## Results

A total of 60 patients met the inclusion criteria for the study. Among them, 27 patients underwent strabismus surgery, while 33 patients received BTX-A injections. At baseline, no significant differences were observed between the two groups with respect to age, sex distribution, BCVA, cycloplegic refractive error, preoperative deviation and visual function (all *P* > 0.05) (Table 1). The age range of participants in BTX-A group was from 8 to 54 years. Two children under the age of 13 required general anesthesia for BTX injections.

**Table 1:** Baseline characteristics and clinical measurements of the study groups

Parameter	Surgery group (n = 27)	BTX-A group (n = 33)	*P* value
Age (years)	23 (6.0 to 56.0)	26 (8.0 to 54.0)	0.397
Gender (M/F)	15/12	16/17	0.586
BCVA			
Right eye	1.0 (1.0 to 1.5)	1.0 (1.0 to 1.5)	0.828
Left eye	1.0 (1.0 to 1.5)	1.0 (1.0 to 1.5)	0.824
SE (D)			
Right eye	− 4.13 ± 3.64 (− 13.13 to 1.25)	− 4.11 ± 2.47 (− 8.00 to 0.50)	0.976
Left eye	− 3.57 ± 3.66 (− 13.25 to 1.25)	− 4.09 ± 2.12 (− 8.25 to 0.25)	0.498
AC/A ratio (Δ/D)	3.42 ± 1.27 (1.50 to 5.50)	2.75 ± 1.39 (2.00 to 5.67)	0.222
Deviation (near, PD)	30 (20.0 to 40.0)	30 (20.0 to 40.0)	0.097
Deviation (distance, PD)	30 (20.0 to 40.0)	30 (20.0 to 40.0)	0.163
Fusion	22/27 (81.5%)	27/33 (81.8%)	0.697
Stereopsis (near)	8/27 (29.6%)	11/33 (33.3%)	0.957
Stereopsis (distance)	10/27 (37.0%)	12/33 (36.4%)	0.915

*BTX-A* = botulinum toxin-A; *M* = male; *F* = female; *BCVA* = best-corrected visual acuity; *PD* = prism diopter, *SE* = spherical equivalent, *AC/A* = accommodative convergence/accommodation

In the surgery group, the median preoperative deviation was 30 PD, with values ranging from 20 to 40 PD. The median muscle recession was 5.0 mm, with a range of 4.0 to 5.5 mm. For the chemodenervation group, the median preoperative deviation was approximately 30 PD (range: 20–40 PD). Among the 33 patients, 20 received a dosage of 2.5 units, and 13 received 5.0 units of BTX-A. No severe or long-lasting complications were reported in either group. Temporary postoperative exotropia occurred in four patients (4/33, 12%) in the chemodenervation group, resolving after a median duration of 6 weeks (range: 4–10 weeks).

**Fig. 1:**
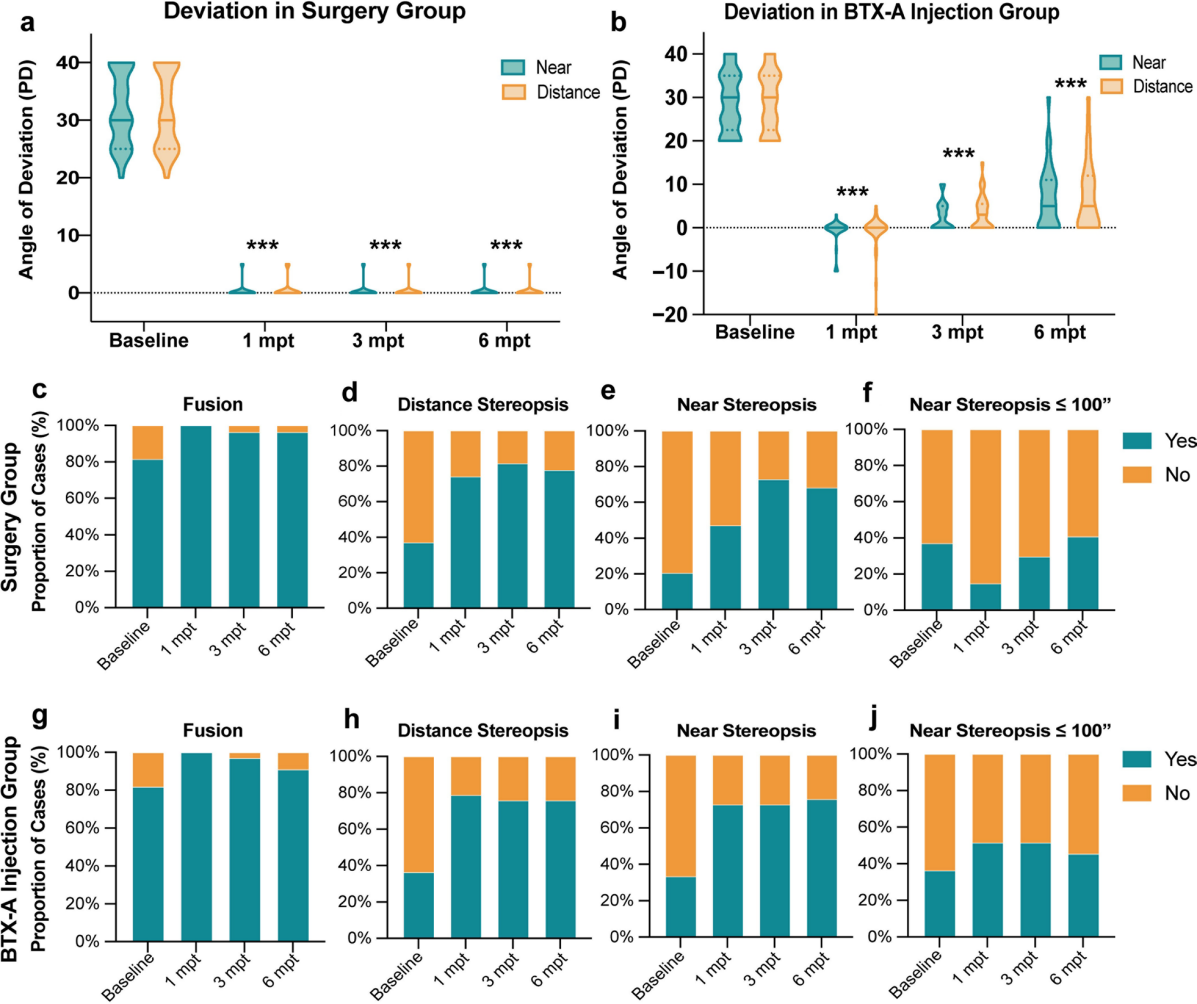
Comparison of motor and sensory outcomes between the two groups. **a–b** Distribution of deviation pre- and post-treatment in the surgery and chemodenervation groups, respectively. **c–f** Percentage of cases with different motor and sensory outcomes in surgery group. **g–j** Percentage of cases with different motor and sensory outcomes in chemodenervation group. BTX-A, botulinum toxin-A; mpt, month post-treatment

Table 2 summarizes the motor and sensory outcomes for all patients. The surgery group achieved a 100% success rate at 1, 3, and 6 months post-treatment. The motor outcome measurements, including the angle of deviation at both near and distance are significantly decreased after surgery (*P* < 0.001 for all, Fig. 1a). In the chemodenervation group, success rates were 85.2% at 1 month, 97% at 3 months, and 69.7% at 6 months post-treatment, respectively (Table 2). Although the angle of deviation at near and distance decreased at 1 month post-treatment in the chemodenervation group, it significantly increased at 6 months post-treatment (*P* < 0.001 for all, Fig. 1b). There were no significant differences in success rates between the two groups at 1 and 3 months post-treatment (*P* > 0.05). However, at 6 months, the surgery group had a significantly higher success rate compared to the chemodenervation group (*P* = 0.001).

**Table 2:** Comparison of motor and sensory outcomes in the surgery and chemodenervation group

Parameter	1 month post-treatment			3 months post-treatment			6 months post-treatment		
	Surgery	BTX-A	*P* value	Surgery	BTX-A	*P* value	Surgery	BTX-A	*P* value
Motor success (near)	27/27 (100%)	23/27 (85.2%)	0.120	27/27 (100%)	33/33 (100%)	–	27/27 (100%)	22/33 (69.7%)	**0.001**
Motor success (distance)	27/27 (100%)	23/27 (85.2%)	0.120	27/27 (100%)	32/33 (97.0%)	1.000	27/27 (100%)	22/33 (69.7%)	**0.001**
Deviation (near, PD)	0 (0 to 5)	0 (− 10 to 3)	0.060	0 (0 to 5)	0 (0 to 10)	**0.001**	0 (0 to 5)	5 (0 to 30)	** < 0.001**
Deviation (distance, PD)	0 (0 to 5)	0 (− 20 to 5)	0.180	0 (0 to 5)	3 (0 to 15)	** < 0.001**	0 (0 to 5)	5 (0 to 30)	** < 0.001**
Fusion	27/27 (100%)	33/33 (100%)	–	26/27 (96.3%)	32/33 (97.0%)	1.000	26/27 (96.3%)	30/33 (90.9%)	0.620
Stereopsis (distance)	20/27 (74.1%)	26/33 (78.8%)	0.668	22/27 (81.5%)	25/33 (75.8%)	0.592	21/27 (77.8%)	25/33 (75.8%)	0.854
Stereopsis (near)	16/27 (59.3%)	24/33 (72.7%)	0.271	22/27 (81.5%)	24/33 (72.7%)	0.425	21/27 (77.8%)	25/33 (75.8%)	0.653
Stereopsis (near, ≤ 100 arcsec)	4/27 (14.8%)	17/33 (51.5%)	** < 0.001**	8/27 (29.6%)	17/33 (51.5%)	0.087	11/27 (40.7%)	15/33 (45.5%)	0.714

*BTX-A* = botulinum toxin-A; *PD* = prism diopter

*P* values in bold indicate statistical significance

In the chemodenervation group, four patients exhibited postoperative exotropia with a median deviation of − 10 PD, and three patients presented with esotropia, showing a median deviation of 5 PD at 1 month post-treatment. By 3 months post-treatment, only one patient demonstrated a deviation of 15 PD of deviation. By 6 months post-treatment, 11 patients developed recurrent esotropia, with median deviations of 13 PD at near (range: 12–30 PD) and 15 PD at distance (range: 12–30 PD) (Table 2). In contrast, in the surgery group, only two patients showed a postoperative deviation of 5 PD at both near and distance at all follow-up points. At the final follow-up, no patients in either group exhibited overcorrection.

Sensory outcomes, including fusion response and stereopsis at both near and distance, significantly improved compared to preoperative values in both groups at 6 months post-treatment (*P* < 0.05 for all, Table 2, Fig. 1c–f). By the final visit, no significant differences in sensory outcomes were observed between the chemodenervation and surgery groups (*P* > 0.05 for all). Notably, at 1 month post-treatment, the success rate for achieving stereopsis at near ≤ 100 arcsec was significantly higher in the chemodenervation group compared to the surgery group (*P* < 0.001).

Logistic regression analysis of clinical characteristics, including age, gender, BCVA, SE, anisometropia, BTX dosage, and preoperative deviation at near and distance, revealed that only anisometropia was associated with BTX injection (*P* = 0.016). Table 3 shows the comparison of motor and sensory outcomes of the patients in the chemodenervation group with different ΔSE values. In this group, anisometropic patients (ΔSE ≥ 1 D) was found to have significantly larger preoperative deviation angles at both near (12.1 ± 4.3 PD) and distance (12.4 ± 4.4 PD) compared to non-anisometropic patients (ΔSE < 1 D; 5.6 ± 7.7 PD at near and 6.2 ± 8.5 PD at distance; *P* = 0.007 and *P* = 0.014, respectively). In this group, non-anisometropic patients (19/25, 76%) showed a higher motor success rate than anisometropic patients (*P* = 0.044). Subgroup analysis revealed that non-anisometropic patients had significantly better sensory outcomes, including stereopsis at near (84%, 21/25) and stereopsis at near ≤ 100 arcsec (56%, 14/25), compared to anisometropic patients (37.5%, 3/8 and 12.5%, 1/8, respectively). No significant differences were observed in fusion or distance stereopsis between groups with different ΔSE values (*P* = 1.000).

**Table 3:** Comparison of motor and sensory outcomes in the chemodenervation group with different anisometropia

Parameter	1 month post-treatment	3 months post-treatment	6 months post-treatment
Motor success (near)			
∆SE < 1 D	21/25 (84%)	25/25 (100%)	19/25 (76%)
∆SE ≥ 1 D	8/8 (100%)	8/8 (100%)	3/8 (37.5%)
*P* value	0.550	–	**0.044**
Motor success (distance)			
∆SE < 1 D	21/25 (84%)	25/25 (100%)	19/25 (76%)
∆SE ≥ 1 D	8/8 (100%)	7/8 (87.5%)	3/8 (37.5%)
*P* value	0.550	1.000	**0.044**
Deviation (near, PD)			
∆SE < 1 D	0 (− 10 to 0)	0 (0 to 10)	3 (0 to 30)
∆SE ≥ 1 D	0 (0 to 3)	3 (0 to 6)	12 (5 to 20)
*P* value	0.272	0.330	**0.009**
Deviation (distance, PD)			
∆SE < 1 D	0 (− 20 to 5)	0 (0 to 15)	3 (0 to 30)
∆SE ≥ 1 D	0 (0 to 3)	3 (0 to 7)	12 (5 to 20)
*P* value	0.470	0.696	**0.015**
Fusion			
∆SE < 1 D	25/25 (100%)	25/25 (100%)	23/25 (92%)
∆SE ≥ 1 D	8/8 (100%)	7/8 (87.5%)	7/8 (87.5%)
*P* value	–	0.242	1.000
Stereopsis (distance)			
∆SE < 1 D	19/25 (76%)	19/25 (76%)	19/25 (76%)
∆SE ≥ 1 D	7/8 (87.5%)	6/8 (75%)	6/8 (75%)
*P* value	0.652	1.000	1.000
Stereopsis (near)			
∆SE < 1 D	21/25 (84%)	20/25 (80%)	21/25 (84%)
∆SE ≥ 1 D	3/8 (37.5%)	4/8 (50%)	3/8 (37.5%)
*P* value	**0.020**	0.170	**0.020**
Stereopsis (near, ≤ 100 arcsec)			
∆SE < 1 D	16/25 (64%)	16/25 (64%)	14/25 (56%)
∆SE ≥ 1 D	2/8 (25%)	0/8 (0%)	1/8 (12.5%)
*P* value	0.101	**0.017**	**0.046**

*SE* = spherical equivalent

*P* values in bold indicate statistical significance

## Discussion

Our study reveals that both incisional strabismus surgery and BTX injection are effective in treating AACE. Surgery provided consistent and stable motor alignment over time, while BTX injections led to earlier sensory success and sustained long-term fusion function. More importantly, we identified that AACE patients with anisometropia > 1 D experienced a higher recurrence rate following BTX injections. These findings provide crucial clinical insights, guiding clinicians in selecting the most appropriate therapeutic approach for patients with AACE, particularly those with anisometropia. To the best of our knowledge, for the first time, this prospective comparative study identifies the risk factors for recurrence following BTX injection in AACE patients.

Our results showed no significant difference in motor success rates between chemodenervation and incisional surgery at 1 and 3 months post-treatment. However, by 6 months, the motor success rate in the chemodenervation group reached 69.7%, confirming its efficacy in treating AACE, albeit lower than the 100% success rate in the surgery group. Similar findings were report by Cheung et al. who found no significant differences between the two treatments at 6 and 12 months in an international multi-center retrospective study, but noted a significantly higher success rate for surgery at 24 months [11]. Despite their longer follow-up period, their cohort included only children. Suwannaraj et al. included both adults and children, highlighting the variability in outcomes based on patient demographics and preoperative characteristics [12]. This discrepancy may be attributed to their participants having a preoperative deviation > 40 PD and only one medial rectus muscle being injected in all chemodenervation procedures. Other studies that observed lower efficacy of BTX-A injections also utilized lower doses, administered unilaterally, and conducted follow-up over only a 6-month period [13].

Previous studies comparing the efficacy of chemodenervation and strabismus surgery in treating AACE have generally found comparable results for both methods. In a retrospective analysis involving 49 patients, Wan and colleagues demonstrated that the efficacy of BTX was not inferior to surgery [14]. Further, a recent meta-analysis of nine studies involving 1,100 participants revealed that although surgery was more effective for various types of comitant esotropia, such as infantile and partially accommodative esotropia, chemodenervation proved equal effective in treating AACE [15]. These results support our findings and highlight the potential of chemodenervation therapy for AACE, underscoring the necessity to advance research on chemodenervation strategies for this condition.

Our study also revealed that the chemodenervation group achieved sensory success earlier than the surgery group, with effects persisting up to 6 months post-treatment. This early success may be attributed to several factors. First, BTX temporarily relaxes the medial rectus by inhibiting acetylcholine release, leading to a rapid reduction in muscle overactivity and facilitating early eye alignment, which places images within the fusion range [16]. Second, studies suggest that BTX may induce long-term changes at the neuromuscular junction, potentially promoting sustained muscle relaxation and alignment [17]. Third, rapid eye alignment helps restore binocular visual function by allowing the brain to reintegrate visual information from both eyes, and thus enhance sensory fusion. Additionally, the stretching effect of the relaxed muscle may contribute to stable alignment, further supporting the early recovery of binocular function [18]. Scott et al. pioneered reporting BTX's efficacy in treating strabismus, noting particularly its substantial benefits in adults with minor horizontal deviations and quick restoration of binocular vision [19]. Similarly, Tejedor and Rodriguez observed that chemodenervation rapidly corrected ocular misalignment and facilitated binocular fusion recovery, with increasing effectiveness with subsequent injections [20]. Cheung et al.’s study supported these observations, reporting a sensory success rate of 70.2% at 6 months post-chemodenervation [11], which closely aligns with our findings.

Although the surgery group demonstrated nearly 100% motor success, some patients exhibited inferior stereopsis despite stable ocular alignment. This discrepancy can be attributed to the fact that motor alignment, while essential, does not always guarantee full restoration of sensory fusion or optimal stereoacuity. Factors such as accommodative convergence, the degree of binocularity, and the timing of visual system adaptation post-surgery may influence stereoacuity [21, 22]. While surgical intervention ensures stable alignment, it does not necessarily result in complete sensory fusion or perfect stereopsis, highlighting the complexity of binocular vision recovery. Additionally, the development of stereopsis may be further influenced by visual training and the brain’s ability to integrate the corrected alignment over time. Future studies are needed to better understand the long-term effects of strabismus surgery on stereopsis.

Although most clinical guidelines suggest that the clinical effects of BTX gradually diminish after 3 to 4 months, animal studies have shown that temporary weakness induced by BTX injections into unilateral horizontal muscles can last from 2 weeks to 8 months [23]. Our study found that, despite a recurrence of strabismus at 6 months post-treatment, patients retained a certain level of binocular visual function, indicating that BTX enhances control for patients with AACE. While the surgical group demonstrated complete alignment from the first month, the BTX-A group exhibited comparable initial correction, which gradually diminished over time as some patients developed residual esotropia. This fluctuation in alignment within the BTX-A group explains the lack of sustained improvement in stereopsis, despite similar initial corrections between the two groups. The success rate of chemodenervation could potentially be higher with multiple injections. Shi and colleagues proposed that treatment can be considered successful after up to three repeat BTX injections [24]. Similarly, Tejedor and Rodriguez reported that the motor success rate significantly improved with the number of injections: 52.9% with one injection, 70.6% with two injections, and 88.2% with three injections in cases of acquired esotropia [25]. Our study focused solely on patients receiving initial treatment; subsequent research has indicated that repeated injections can further improve control of eye position in AACE. (unpublished data).

Previous studies predominantly employed retrospective designs and rarely included measurements of refractive status [11, 12, 14, 26]. This study is the first to incorporate cycloplegic refraction results, identifying anisometropia as a potential risk factor for recurrence in AACE patients treated with BTX. The underlying mechanism involves an imbalance in the binocular accommodative-convergence function. Patients with anisometropia experience different refractive states between their eyes, requiring varied accommodative efforts during visual fixation [27]. This disparity can decouple accommodative and convergence processes, particularly in cases of pronounced anisometropia, which exacerbate instability within the binocular visual system [28]. Consequently, while BTX injections may initially correct ocular misalignment, they often fail to sustain long-term binocular fusion. The misalignment between accommodation and convergence likely contributes to strabismus recurrence as the neuromuscular blockade from BTX diminishes [29]. It has been reported that anisometropia disrupts convergence responses, adversely affecting the long-term outcomes of strabismus treatment [30]. Therefore, clinicians should carefully consider the degree of anisometropia when selecting treatment strategies for AACE patients to optimize long-term therapeutic outcomes.

Additional potential risk factors that may affect the efficacy of BTX-A treatment include the prolonged duration from the onset of esotropia to treatment, patient age, and large preoperative deviation angles. Sheth and colleagues found that patients with acute comitant esotropia who underwent surgery 20.6 months after onset had a lower success rate compared to those operated on at 10.9 months post-onset [31]. However, their study lacked data on stereopsis. Yagasaki et al. discovered that patients undergoing strabismus surgery more than 12 months after onset have poorer stereoacuity [32]. Previous research also indicated that larger deviation angles, especially at near, are more challenging to correct with BTX [33]. Scott et al. reported that the maximum correction range for strabismus treated with BTX is 40 PD [19]. However, our study did not identify other risk factors affecting the efficacy of BTX treatment beyond anisometropia due to the smaller sample size and the timely interventions administered.

There are several limitations within this study. The small sample size is insufficient for generalizing findings to diverse populations or varying severities of AACE, and thus further studies with larger cohorts are necessary for conclusive clinical evidence. Additionally, the follow-up period was limited to 6 months, preventing an assessment of the long-term sustainability of treatment effects. Treatment allocation based on patient preference rather than random assignment can introduce selection bias, affecting the internal validity of the study. The lack of standardized dosage guidelines for BTX may result in variability in treatment efficacy, thereby complicating comparisons of its effectiveness in a broader clinical context. These factors emphasize the necessity for future studies with larger samples, extended follow-up periods, and more rigorous controls to thoroughly evaluate the long-term benefits and comparative effectiveness of these treatments for AACE.

## Conclusions

In conclusion, this study compares the efficacy of traditional incisional surgery and BTX injections in managing AACE, demonstrating distinct advantages and limitations associated with each treatment modality. The success rate of surgery remains consistently high across various follow-up intervals. In contrast, while BTX injections are initially effective, they exhibit a decline in success rates over a 6-month period. Nonetheless, it provides reliable and effective treatment for enhancing stereopsis. This study emphasizes anisometropia as a significant factor affecting outcomes with BTX, underscoring the necessity for tailored treatment strategies. Further studies are essential to refine BTX dosages and develop comprehensive, patient-centered outcome measures. This approach would enhance treatment precision and potentially expand the application of BTX in managing AACE.

## Data Availability

Due to institutional policy, the datasets are not publicly available. However, they are available from the corresponding author upon reasonable request and with permission from the institution.

## Abbreviations

AACE: Acute acquired comitant esotropia
BCVA: Best-corrected visual acuity
BTX-A: Botulinum toxin-A
PD: Prism diopter
SE: Spherical equivalent
